# Editorial: Expert opinions in neurocognitive aging and behavior: neural rehabilitation for elder people

**DOI:** 10.3389/fnagi.2023.1263417

**Published:** 2023-08-02

**Authors:** Ping Zhou, Yingchun Zhang, Sheng Li

**Affiliations:** ^1^School of Rehabilitation Science and Engineering, University of Health and Rehabilitation Sciences, Qingdao, Shandong, China; ^2^Department of Biomedical Engineering, University of Houston, Houston, TX, United States; ^3^Department of Physical Medicine and Rehabilitation, University of Texas Health Science Center at Houston, Houston, TX, United States

**Keywords:** neuromotor function, aging, rehabilitation, opinion, perspective

As advanced age affects body organs and systems, older people are more vulnerable to neurological conditions or disorders. This Research Topic has collected articles that provide opinions and perspectives related to the field of neural rehabilitation for the elderly, particularly on aspects such as neuromotor function, rehabilitation, and exercise.

With aging, older adults experience significant declines in cognitive function. Tao et al. discussed cognitive-motor dual task training (CMDT) for the prevention and treatment of aging-related cognitive impairment. Compared to a single training modality, CMDT appears to be a more effective intervention for improving cognitive function and brain structure, due to its synergistic and complementary nature. Therefore, CMDT offers a promising intervention strategy for aging-related cognitive impairment. Although it has a good prospective application, the standardization of its applications and the underlying neurophysiological mechanisms require further investigation.

In line with the “Exercise is Medicine” initiative advocated by the American College of Sports Medicine and the American Medical Association, Li et al. discussed the role of exercise in healthcare, particularly the importance of exercise in the prevention and treatment of a range of age-associated diseases or health conditions. Several modules and principles for implementing exercise as a form of medicine were proposed, with considerations of appropriate intensity and potential side effects. Given that the lack of exercise is a global health concern, “Exercise is Medicine” is a timely call to promote the involvement of people in health or exercise programs, including the elderly and individuals who experience a disability.

Quantitative physiological measurements are needed for successful rehabilitation. Gong et al. discussed the significance of combining electrical impedance myography (EIM) with other commonly used quantitative methods, such as medical imaging, electromyography (EMG), and myotonometry, etc., to assess muscle alterations after stroke. Different combinations were suggested to evaluate morphological and functional changes in paretic muscle. Multimodal parameters resulting from different techniques can facilitate a comprehensive muscle evaluation, which is important for understanding the physiological mechanisms underlying dysfunction and rehabilitation.

With the goal of long-term neurorehabilitation after stroke, Zhou et al. presented their opinions on automated theranostics, focusing on technical automation in neuro-behavioral measurements, rehabilitation treatments, and coordination of healthcare resources. Recent progress, existing gaps, and future directions were discussed in depth. In addition, other relevant factors such as cross-disciplinary education and equity for underdeveloped areas were also considered to promote the application of automatic theranostics. These multifaceted advances collectively help implement rehabilitation theories and clinical expertise into automation infrastructures, thus facilitating long-term neurorehabilitation.

Hu et al. discussed aging-related changes in speech production, which is a complex neuromotor behavior involving multiple body systems. While the majority of previous studies in this field have mainly examined the acoustic changes, the opinion article focused on the neuromotor control of speech production with aging, which has not been extensively studied. Multiple factors involved in the aging-related neuromotor control of speech production were discussed, including laryngeal physiology, brain structure and function, higher-order cognitive functions, and sex-aging interactions. Understanding the effects of aging on neuromotor control of speech production is important for the appropriate treatment of motor speech disorders that frequently occur with advanced aging.

Finally, Salvalaggio et al. presented a protocol study designed to investigate the effect of rehabilitation treatment in a group of stroke survivors. Cross-modality protocols were proposed to develop a multidimensional predictive model of sensorimotor recovery related to stroke upper limb rehabilitation, characterized by integrating physiological and imaging techniques with clinical and cognitive assessment, rehabilitation dose and biological variables in stroke patients undergoing a period of intensive rehabilitation. The described study will generate a rich multi-modal dataset in stroke patients. The prospective protocols, if validated, can help promote individualized rehabilitation targeting the best possible outcome.

We hope that this Research Topic will help to promote thoughts and perspectives on recent technological advances, latest discoveries, current challenges, and future directions specifically in the field of neural rehabilitation for the elderly.

## Author contributions

PZ: Writing—original draft. YZ: Writing—review and editing. SL: Writing—review and editing.

